# Improving Doppler Radar Precipitation Prediction with Citizen Science Rain Gauges and Deep Learning

**DOI:** 10.3390/s25123719

**Published:** 2025-06-13

**Authors:** Marshall Rosenhoover, John Rushing, John Beck, Kelsey White, Sara Graves

**Affiliations:** Information Technology and Systems Center, University of Alabama in Huntsville, Huntsville, AL 35899, USA; john.rushing@uah.edu (J.R.); john.beck@uah.edu (J.B.); kelsey.white@uah.edu (K.W.); sara.graves@uah.edu (S.G.)

**Keywords:** radar precipitation estimation, radar-rain gauge rainfall accumulation, citizen science, deep learning, rainfall accumulation, radar calibration, real-time weather prediction

## Abstract

Accurate, real-time estimation of rainfall from Doppler radars remains a challenging problem, particularly over complex terrain where vertical beam sampling, atmospheric effects, and radar quality limitations introduce significant biases. In this work, we leverage citizen science rain gauge observations to develop a deep learning framework that corrects biases in radar-derived surface precipitation rates at high temporal resolution. A key step in our approach is the construction of piecewise-linear rainfall accumulation functions, which align gauge measurements with radar estimates and allow for the generation of high-quality instantaneous rain rate labels from rain gauge observations. After validating gauges through a two-stage temporal and spatial consistency filter, we train an adapted ResNet-101 model to classify rainfall intensity from sequences of surface precipitation rate estimates. Our model substantially improves precipitation classification accuracy relative to NOAA’s operational radar products within observed spatial regions, achieving large gains in precision, recall, and F1 score. While generalization to completely unseen regions remains more challenging, particularly for higher-intensity rainfall, modest improvements over baseline radar estimates are still observed in low-intensity rainfall. These results highlight how combining citizen science data with physically informed accumulation fitting and deep learning can meaningfully improve real-time radar-based rainfall estimation and support operational forecasting in complex environments.

## 1. Introduction

Accurate, real-time rainfall estimates at fine spatial and temporal scales are essential for modern weather forecasting, flood warning, and emergency response systems. Yet predicting instantaneous surface precipitation rates (SPRs) from Doppler radars remains challenging: radar signals sample precipitation aloft—often several hundred meters above the ground—while beam widening with distance and variable overlap between neighboring radars can introduce vertical and horizontal biases into raw SPR fields [1,2]. In addition, complex terrain and stratified precipitation regimes further degrade radar-based estimates, leading to under or over-prediction of localized precipitation events.

To correct systematic biases in radar-derived precipitation fields, meteorological agencies generate quantitative precipitation estimates (QPEs)—retrospective rainfall totals that blend data from rain gauges, satellite observations, radar mosaics, and numerical models. These products are widely used to post correct SPRs by providing an absolute rainfall reference, improving accuracy at hourly to daily timescales. However, QPEs are produced through post-event analysis and are subject to latencies of 50–120 min, as seen in systems like NOAA’s Multi-Radar Multi-Sensor (MRMS) platform [2,3,4]. They also suffer from persistent gauge coverage gaps, particularly in rural and mountainous regions. As a result, while QPEs serve as the primary correction mechanism for radar-based rainfall estimates, they are not available in real time—leaving operational radar SPR products largely uncorrected during fast-evolving events.

Citizen science networks offer a promising avenue to address the lack of real-time correction for radar-based precipitation estimates. By engaging volunteers to collect and report local rainfall, programs such as CoCoRaHS, GLOBE, and Weather Underground have created dense, geographically distributed sensor networks that complement traditional gauge infrastructure. These networks include backyard rain gauges, school-based instruments, and low-cost DIY sensors that help fill spatial gaps in rural, mountainous, and under-instrumented regions. When appropriately quality-controlled, citizen observations have been shown to support radar bias correction, validate QPE products, and improve climatological assessments at hourly to daily timescales [5,6,7,8,9,10]. Recent studies have further demonstrated their potential to enhance aggregate rainfall estimates, correct radar regional biases at multi hour intervals, and initialize numerical weather models [11,12,13]. However, these efforts have not extended to real-time correction of radar SPRs at high temporal resolution.

Recent advances in deep learning offer a powerful framework to address these real time estimation challenges. Convolutional neural networks (CNNs), in particular, can ingest raw radar reflectivity fields, identify evolving storm structures, and predict high-resolution precipitation rates in near real time. Unlike traditional statistical or physically based models, CNNs learn complex spatiotemporal patterns directly from data, enabling them to generalize across varied climatologies and sensor platforms. Early applications—such as optical-flow-based CNNs and U-Net variants—have shown skill at short-term rainfall nowcasting, often outperforming persistence and classical extrapolation methods in both accuracy and critical-event detection [14,15].

In this work, we propose a deep learning approach that leverages historical citizen science rain gauge data to improve real-time radar-based precipitation estimates at two minute resolution. Rather than relying on live gauge inputs—which are often delayed, sparse, or inconsistent—we train a model on archived crowd sourced observations to predict instantaneous rain rates at gauge locations, using two minute Doppler radar fields. This approach both reduces latency and increases the spatial density of ground truth data, addressing key limitations of traditional rain gauge and QPE systems. Inspired by the classification framework of Agrawal et al. [16], we formulate rain rate prediction as a multi-class image classification task, using thresholds for trace, light, and moderate precipitation intensities.

A central challenge in this approach is that citizen-science rain gauges do not report instantaneous rain rates—they provide only accumulated totals at discrete observation times. Meanwhile, radar data is available every two minutes but is spatially averaged and often noisy near the surface. To train a model capable of classifying precipitation intensity from radar images at gauge locations, we must first generate high temporal resolution surface rain rate labels. We address this by reconstructing continuous accumulation functions that integrate the temporal structure of SPR fields with the discrete accumulation measurements from the gauges. Differentiating these functions yields two minute surface rain rate estimates that are consistent with both data sources, enabling label generation for supervised learning.

The key contributions of this work are as follows:

1. We introduce a piecewise linear accumulation fitting method that reconstructs rain accumulation functions by aligning sequences of radar-derived SPR fields with discrete gauge measured accumulations, enabling the generation of high-resolution surface rain rate labels needed for supervised learning.
2. We develop a two-stage data validation process that verifies the reliability of citizen science rain gauge measurements without requiring prior knowledge of individual station calibration, siting, or instrumentation.
3. We train a deep learning model to learn localized radar calibration corrections and demonstrate that it substantially improves precipitation classification accuracy relative to NOAA’s operational Doppler radar SPR products, achieving significantly higher macro precision, recall, and F1 scores within observed spatial regions.

By integrating dense citizen-science gauge measurements with state of the art deep learning, we demonstrate a viable pathway toward real-time high-resolution precipitation predictions that can be useful for operational forecasting and flood-warning systems.

## 2. Data Setup

The Hawaiian Islands present a uniquely challenging environment for validating radar-based precipitation estimates. Their steep topography, complex microclimates, and exposure to trade winds, mid-latitude systems, and tropical cyclones create some of the most spatially diverse and extreme rainfall patterns on Earth [17]. This rugged terrain also obstructs ground-based Doppler radar coverage, causing beam blockage, overshooting, and substantial gaps in surface precipitation visibility. Accurately capturing rainfall across such a dynamic landscape requires observations that are both frequent and geographically dense. To address these challenges, we combined Doppler surface precipitation rates with a dense network of citizen science rain gauge observations. Our study spans four years, from March 2020 through March 2024, covering a broad range of weather conditions and rainfall intensities.

### 2.1. Radar

NOAA provides SPR estimates for Hawaii with a temporal resolution of two minutes and a spatial resolution of 500 m. These estimates are derived from radar reflectivity data and are adjusted to better represent near-surface precipitation. Key corrections include identifying the melting layer using atmospheric temperature profiles and accounting for evaporative losses below the cloud base [1].

**Figure 1:**
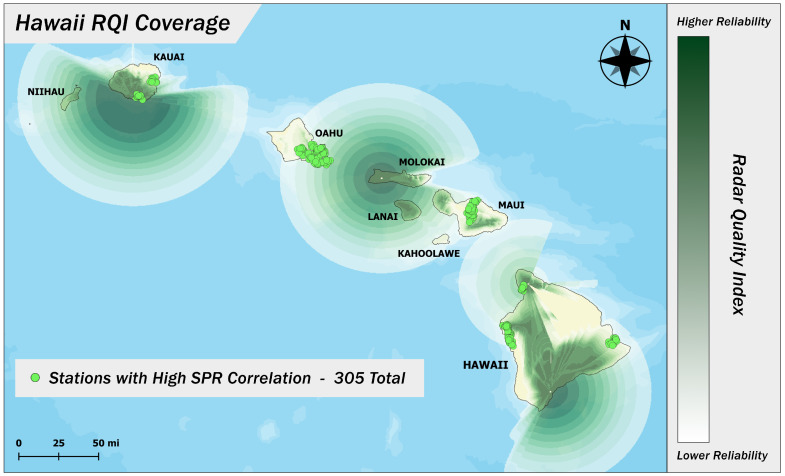
Locations of validated personal weather stations with respect to the radar’s RQI.

Despite these refinements, radar-based precipitation estimates are still subject to several sources of uncertainty. Beam blockage by terrain, signal attenuation during heavy precipitation, and reflectivity biases can all affect the accuracy of the radar signal reaching the surface. To help characterize these limitations, NOAA also provides a Radar Quality Index (RQI), which quantifies the expected reliability of radar estimates at each location [18]. The RQI ranges from 0 (poor quality) to 1 (high quality) and reflects factors such as beam geometry and signal path obstructions. It does not correct for these issues but instead offers a spatially explicit measure of confidence in the underlying precipitation estimates. A sample RQI field for Hawaii during a precipitation event is shown in Figure 1.

Systematic biases in radar estimates are well documented, particularly over longer accumulation periods where averaging reveals consistent underestimation during high-intensity events (due to beam overshoot and attenuation) and overestimation during light rain (due to reflectivity biases and minimum detection thresholds) [19]. These patterns inform the generation of post-processed QPE products and traditional bias correction schemes.

At finer, two-minute timescales, however, the radar–gauge relationship becomes substantially more variable, and the spatial and temporal patterns of bias observed at coarser resolutions often break down. Instantaneous errors are frequently treated as stochastic or uninformative—dismissed as noise [20]. Yet this assumption overlooks the possibility that radar errors at short timescales may contain learnable patterns—raising the need to examine how these high-frequency estimates relate to ground-based observations.

### 2.2. Citizen Science–Rain Gauges

To combine radar predictions with ground-based observations, we leveraged rainfall data from 704 personal weather stations (PWSs) sourced from the Weather Underground network. These stations are distributed unevenly across Hawaii, with varying periods of activity—some reporting data for only a few months, others remaining online throughout the entire study period. PWSs commonly use tipping-bucket mechanisms or optical sensors to measure precipitation, converting the collected volume into accumulated depth over fixed time intervals. As a result, the data represents incremental accumulations over time.

**Figure 2:**
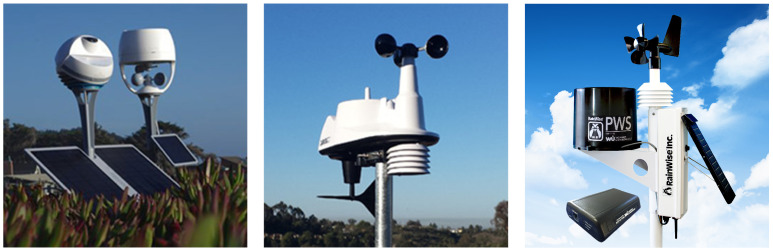
The diversity of personal weather stations, reflecting variations in design and deployment [21].

Because the reports of PWSs data differs from the instantaneous nature of radar estimates, pre-processing is required to combine the two data streams. However, additional challenges arise from the fact that these stations are privately operated and lack professional standardization. Their data can vary significantly in quality due to differences in hardware, siting conditions, calibration practices, and maintenance levels. A visual sample of the diversity in PWS designs and deployments is shown in Figure 2.

Due to this variability, no initial assumptions were made about the accuracy or sensitivity of any individual station. Instead, in the next section, each station is treated as a potentially useful but unverified source of observation, requiring independent validation before use in model training or evaluation.

### 2.3. Generating Rainfall Accumulation Functions

Radar and PWS data differ both in temporal resolution and measurement type: a radar provides two-minute estimates of surface precipitation rates averaged over 500 m grid cells, while PWS gauges report accumulated rainfall at irregular, typically five-minute intervals. To derive high-resolution rain rate labels suitable for supervised learning of our model, we reconstruct continuous rainfall accumulation functions at each gauge location by combining the temporal structure of radar estimates with the total accumulations reported by the gauges. Each gauge observation acts as an integral constraint on the accumulation function, and the radar provides the basis for estimating how rainfall varied between observations. This reconstruction process involves four main steps:

1. Gauge Sensitivity Analysis: For each station, we calculate its minimum detectable accumulation increment, which constrains how accumulation can change between readings.
2. Bound Estimation: Using this sensitivity, we define upper and lower bounds on possible true accumulation between each pair of gauge observations, ensuring that reconstructed functions remain physically plausible.
3. Radar-Guided Interpolation: We use radar-derived rain rates to approximate how rainfall was distributed within each interval.
4. Constraint Adjustment: Finally, we scale the radar-based estimates linearly within each interval so that their integrated total is within the gauge constraints.

**Figure 3:**
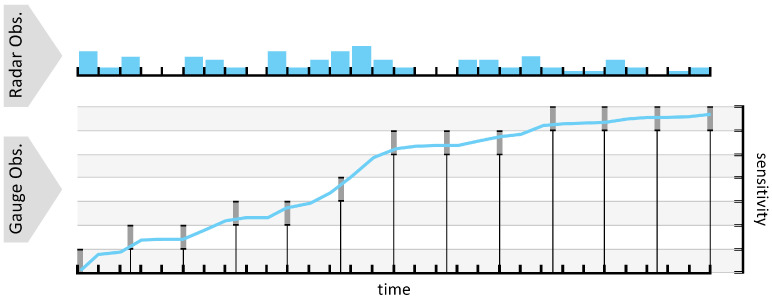
(**Top**) Two minute radar-derived instantaneous precipitation rates. (**Bottom**) Gauge discrete reporting instants with measurement-sensitivity bounds (gray lines), and the fitted continuous accumulation function (blue line). This illustrates how radar temporal patterns and gauge integral constraints are integrated to generate high-resolution surface rain-rate labels.

Executing these steps produces a piecewise-linear accumulation curve that exactly satisfies each gauge’s observed totals while following the high-frequency variability captured by the radar. Figure 3 shows this reconstruction for a representative gauge.

#### 2.3.1. Gauge Sensitivity Analysis

Rain gauges in PWSs report rainfall accumulation as a series of discrete values, each reflecting a stepwise increase rather than a continuous measurement. Each step corresponds to a fixed minimum detectable increment, defined by the gauge sensitivity *m*. This sensitivity determines the resolution at which the gauge can detect changes in rainfall, shaping how accumulation is quantified over time. To accurately reconstruct a continuous accumulation function from these discrete reports, we must first estimate *m* for each individual gauge. Since PWS devices are not standardized, the sensitivity must be inferred directly from the data itself.

We model each recorded observation as a rounded-down version of the true cumulative rainfall:

$$O_{i}=m\cdot \left\lfloor \frac{A_{i}}{m}\right\rfloor$$

where $A_{i}$ is the true but unknown cumulative rainfall at observation *i*, *m* is the gauge sensitivity, and $O_{i}$ is the reported value. This equation captures the fact that accumulation is only reported in discrete multiples of *m*. To estimate *m*, we compute the smallest nonzero increase between successive observations in the gauge’s historical time series:

$$m=\min\{O_{i+1}-O_{i}\;|\;O_{i+1}>O_{i}\}$$

This assumes that, across a sufficiently long record, at least one observed increment reflects the smallest possible step size.

#### 2.3.2. Bound Estimation

Once the gauge sensitivity *m* is known, we can impose physically meaningful constraints on the true rainfall accumulation, both at observation points and over the intervals between them. Although rain gauges report accumulated rainfall at discrete time steps, the true accumulation is a continuous quantity that is only partially observed due to quantization.

Given the gauge sensitivity, we can bound the true accumulation for a reported observation value using the quantization model described previously:

(1) $$\begin{array}{c} O_{i}\;\leq \;A_{i}\;<\;O_{i}+m, \end{array}$$

This range defines the set of possible true accumulation values consistent with a single gauge observation. Given two consecutive observations i−1 and *i*, and the fact that rainfall is non-negative, it follows that $A_{i}\geq A_{i-1}$ and therefore $O_{i}\geq O_{i-1}$. This relationship forms the basis for connecting discrete gauge readings to the underlying rainfall process. Specifically, the change in accumulated rainfall between observations can be expressed as the integral of the true instantaneous rain rate r(t) over the interval

(2) $$\begin{array}{c} A_{i}=A_{i-1}+\int_{i-1}^{i}r\left(t\right)\;dt \end{array}$$

This equation links the cumulative accumulation at two time points via the continuous rain rate function. By combining this with the quantization bounds at both ends of the interval, we derive a constraint on the total rainfall over the interval

(3) $$\begin{array}{c} O_{i-1}\;\leq \;A_{i-1}+\int_{i-1}^{i}r\left(t\right)\;dt\;<\;O_{i}+m, \end{array}$$

This inequality defines a physically valid range for the true accumulation at any time between and at observations, constrained jointly by the rain gauge observation resolution and the non-decreasing nature of rainfall.

#### 2.3.3. Radar-Guided Interpolation

Although radar measurements are influenced by spatial averaging, beam geometry, and environmental conditions, they still capture useful temporal variation in the rainfall intensity. While not perfect measurements of the true instantaneous rain rate r(t), radar-derived SPR provides a noisy signal that reflects underlying precipitation patterns. We treat this time series as a discretized sampling of r(t), allowing us to approximate the integral of rain rate over a gauge interval. Specifically, we estimate the total accumulation between two gauge observations as

(4) $$\begin{array}{c} A_{r}\left(i-1,i\right)=\sum_{t\in [i-1,i]}Radar\left(t\right)\Delta t\;\approx \;\int_{i-1}^{i}r\left(t\right)\;dt \end{array}$$

Here, Radar(t) is the radar-estimated rain rate at time t, and Δt is the radar sampling interval. This estimated radar accumulation, $A_{r}\left(i-1,i\right)$, provides a plausible approximation of how rainfall may have been distributed over the interval.

#### 2.3.4. Constraint Adjustment

Despite capturing high resolution temporal variation, radar-derived accumulation totals often do not match the accumulation recorded by the gauge. This mismatch arises from spatial averaging in radar measurements, 500 m rain rate averaging, and noise from the measurements. As a result, the radar-estimated accumulation $A_{r}\left(i-1,i\right)$ may fall outside the valid bounds defined in Equation (3), violating physical constraints.

To resolve this, we apply a linear adjustment that rescales the radar-derived accumulation to match the gauge-observed total over the interval. We compute the scaling ratio as the difference of rain gauge accumulation values divided by the difference in radar accumulation values:

(5) $$\begin{array}{c} \mathrm{Ratio}\left(i\right)=\frac{O_{i}-O_{i-1}}{A_{r}\left(0,i\right)-A_{r}\left(0,i-1\right)} \end{array}$$

This ratio corrects the total radar accumulation over the interval so that it aligns exactly with the observed accumulation from the gauge. Using this ratio, we construct a piece-wise linear approximation of the cumulative rainfall between [i−1,i] for any intermediate time t within the interval i−1≤t≤i. The adjusted accumulation function $\tilde{A}$ is defined as

(6) $$\begin{array}{c} \tilde{A}\left(t\right)=O_{i-1}+\left(A_{r}\left(t,0\right)-A_{r}\left(i-1,0\right)\right)\times \mathrm{Ratio}\left(i\right) \end{array}$$

Here, $A_{r}\left(0,t\right)$ is the radar-estimated cumulative rainfall from the start up to time t, and the term in the parentheses captures the radar accumulation within the current interval. This scaling ensures consistency at the boundaries:

$$\begin{array}{c} \tilde{A}\left(i-1\right)=O_{i-1},\;\tilde{A}\left(i\right)=O_{i} \end{array}$$

The final accumulation function preserves radar-inferred variability while ensuring consistency with the gauge reported totals. Additional implementation details, edge case handling, and limitations of this method are provided in Appendix A.

### 2.4. Identifying Reliable Gauges

Not all PWS rain gauges produced reliable accumulation functions when compared to nearby stations or corresponding radar observations. To filter out inconsistent or inaccurate stations, we developed a two-step validation process. For each gauge, we generated both a rain-gauge-based accumulation curve (6) and a radar-derived accumulation curve (4) at the same location. Because radar estimates can misrepresent absolute rainfall totals, we evaluated their consistency by computing the temporal correlation between the normalized accumulation curves over a day, emphasizing the timing and progression of rainfall. A station was considered valid if it achieved a Pearson correlation coefficient above 65% with the radar-derived accumulation curve across all its observations.

**Figure 4:**
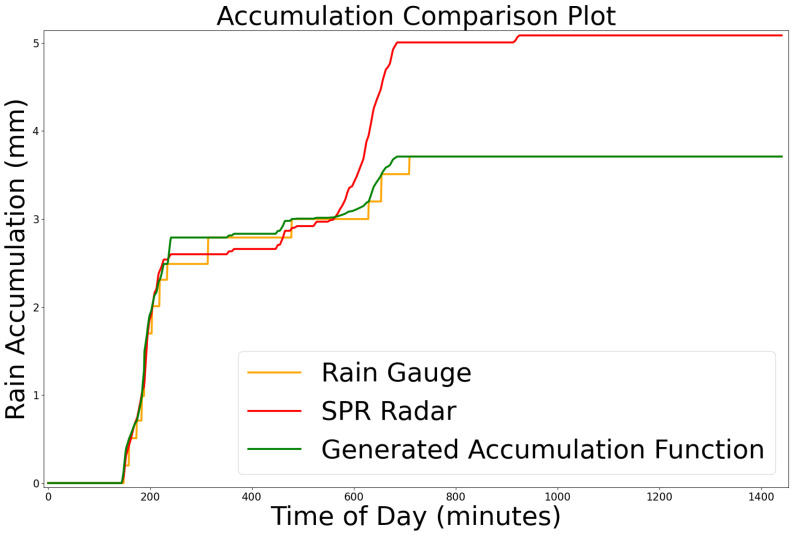
The generated accumulation function, $\tilde{A}$, with a Pearson correlation coefficient of 98.4% along with the original rain gauge data and the accumulation function from the Doppler radar.

If a station failed this radar-based criterion, we then checked whether it had any neighboring stations within a three-kilometer radius that were active during the same time period. If the gauge’s accumulation curve showed a Pearson correlation above 65% with at least one nearby station, it was still accepted as valid—on the premise that its measurements were consistent with local rainfall patterns even if they diverged from radar estimates. Figure 4 shows an example accumulation function that passed the validation process.

We selected the 65% correlation threshold based on the empirical distribution of Pearson correlations between each gauge’s reconstructed accumulation function and the corresponding radar-derived curve. This analysis revealed a broad cluster of stations with moderate-to-high correlation values (typically above 65%), indicating general agreement in temporal rainfall patterns. Below this level, the distribution became increasingly random, suggesting a breakdown in the reliability of the radar–gauge alignment. This inflection point provided a practical threshold for distinguishing broadly consistent stations from those with erratic or untrustworthy behavior. While the 65% cutoff is not an absolute measure of reliability and may occasionally exclude well-performing gauges or admit marginal ones, it struck a balance between filtering out clearly problematic stations and preserving valuable spatial coverage. Stations that failed both validation criteria, or lacked nearby comparisons, were excluded from further analysis. After filtering, we retained 305 validated PWSs across nine spatial clusters, as shown in Figure 1.

### 2.5. Radar-Rain Gauge Dataset Creation

Using the validated stations, we obtained two-minute instantaneous rain rates by taking the derivative of the rain gauge accumulation functions, aligning with the temporal resolution of NOAA’s SPR product. These derived rain rates were categorized into four classes: [0, 0.1), [0.1, 1.0), [1.0, 2.5), and [2.5, *∞*) millimeters per hour (mm/h), allowing us to frame the prediction task as a multi-class classification problem.

For each rain gauge label, we aligned a twenty-minute sequence of radar frames leading up to the observation time. Each sequence was cropped to a 16 × 16 km region centered on the rain gauge location, capturing relevant spatial and temporal context. These sequences serve as the model inputs, while the corresponding rain class provides the target labels. Overall, the dataset contains more than 1.75 million labeled rain classification events.

To structure the dataset for training and evaluation, we grouped stations into spatial clusters based on their island and location. For each cluster, we computed the average RQI over the entire study period. Based on the average RQI, clusters were categorized as high (RQI > 0.7), moderate (0.4 < RQI ≤ 0.7), or low (RQI ≤ 0.4) radar quality.

In designing the testing strategy, we aimed to evaluate the model’s ability to perform both local and general radar calibration. First, to assess local calibration, we constructed a test set comprising 30% of rain events from all clusters except those on Maui. This set includes clusters spanning the full range of RQI values. Strong performance on this subset would indicate that the model can learn localized radar corrections in regions seen during training, regardless of radar quality. It would also suggest that, despite low radar confidence in certain areas, there are consistent patterns in the data that can be exploited to improve predictions in low RQI regions. Second, to assess general calibration, we held out all stations from Maui as a completely unseen test set. Since Maui primarily falls into the low RQI category and was excluded from training, strong performance here would demonstrate the model’s ability to generalize its learned radar corrections to new, unseen regions. It would also suggest that, independent of RQI, there are broader, learnable patterns in the radar data that the model can capture. Together, these two test sets enable an assessment of both localized adaptation and broader generalization in radar-based rainfall prediction. Table 1 summarizes the spatial clusters, their corresponding islands, station counts, average RQI, number of rain events, and test data allocation.

## 3. Experiments and Results

Accurate rainfall estimation requires models that not only capture fine-scale spatial structures but also operate with the speed necessary for real-time forecasting. We therefore built a precipitation classification network on the ResNet-101 backbone [22], whose deep residual connections let it train very deep CNNs without vanishing gradients and capture multi-scale spatial features critical for identifying rainfall signatures. Preliminary tests with smaller variants (ResNet-18 and ResNet-50) underfit the highly localized and diverse rain patterns across Hawaii, motivating our choice of the deeper ResNet-101.

Compared to sequence-based models such as ConvLSTM networks [14], ResNet-101 also offers substantial computational advantages. By operating on fixed-length radar input sequences as stacked image channels, the ResNet approach reduces inference latency and memory overhead, making it far more practical for applications requiring fast prediction turnaround, such as flash-flood warning systems. Additionally, unlike recurrent architectures, which impose a strict sequential structure on the input data, the channel-stacking approach in ResNet allows the model to learn more flexible temporal dependencies. This is particularly beneficial for radar data, which often exhibits noisy fluctuations and non-smooth temporal dynamics due to beam blockage, atmospheric interference, and rapid shifts in precipitation intensity. Enforcing strict temporal continuity in such conditions may cause recurrent models to overfit to short-term variability while missing broader, cross-temporal patterns. In contrast, stacking frames as parallel input channels enables the model to extract both local and long-range features across time without assuming a strict progression, leading to more robust learning under uncertain and discontinuous input conditions.

To adapt ResNet-101 for our radar data, we replaced its standard RGB stem with two sequential 3 × 3 convolutions, each followed by batch normalization and SiLU activation, preserving fine spatial detail from 500 m SPR grids. We retained the rest of the ResNet-101 architecture and appended a linear classification head that outputs logits for the four precipitation categories, matching the thresholds defined previously.

### 3.1. Training Procedure

We trained on stacks of consecutive radar SPR maps centered on each validated gauge, using as ground truth the gauge derived class label at the final timestep. Labels come exclusively from the reconstructed accumulation functions (6), so the model implicitly learns to correct radar biases relative to those gauges. We used the Adam optimizer with a batch size of 128, learning rate 0.0002, and early stopping (patience = 50 epochs). The data were split 70/30 into training/validation, and we ran three independent training trials.

### 3.2. Evaluation Procedure

We report precision, recall, and macro-averaged F1 for each precipitation class, averaged over three independent training runs. Precision measures the proportion of correct predictions among all instances predicted for a given class, indicating how reliable the model’s positive predictions are. Recall quantifies the proportion of actual instances of a class that the model correctly identifies, reflecting its ability to detect relevant events. The F1 score, defined as the harmonic mean of precision and recall, balances these two metrics, and we use macro-averaging to ensure equal weight is given to each class regardless of class frequency. As a baseline (“Radar”), we bin the raw NOAA’s radar SPR estimate at each gauge location into the same four classes. Comparing our model against the SPR baseline highlights the difference between the current technology and the value added by our model. All final results are computed on the held out test sets drawn from weather events unseen during training.

### 3.3. Generalization on Observed Spatial Clusters

The model’s performance across observed spatial clusters, achieving macro F1 scores above 0.91, is noteworthy not just for its accuracy but for what it reveals about its ability to learn radar error correction. As mentioned before, NOAA’s SPR estimates suffer from well-documented distortions that can either suppress or exaggerate rainfall rates. For instance, in the [0.1, 2.5) mm/h range, the radar often overstates moderate rain by interpreting high-altitude echoes that never reach the ground as surface rainfall. That our model improves F1 scores by more than 0.4 points in these categories suggests it has learned localized mechanisms of SPR misrepresentation. By leveraging the 20 min temporal sequence of SPR fields, the model appears to extract spatiotemporal patterns—such as storm evolution, echo persistence, and surrounding structure—that help infer surface-level precipitation more accurately than any single SPR snapshot allows.

Importantly, these gains are consistent across regions with both high and low RQI, suggesting that the model does more than rely on high-confidence radar input. Instead, it learns region-specific error signatures and contextual cues that improve classification regardless of RQI. This indicates that, despite degraded radar quality, there are still consistent spatiotemporal patterns in SPR fields that can be exploited—patterns that may not be obvious but are learnable. The full class-wise results are summarized in Table 2.

### 3.4. Generalization to Unseen Spatial Clusters

The model’s performance on the unseen Maui test set—achieving a macro F1 score of 0.519—indicates that it generalizes only partially beyond the regions it was trained on. While it slightly outperforms the radar baseline overall and improves classification for light and moderate rainfall, it fails to deliver consistent gains in heavier rainfall categories, where performance is comparable to or slightly below the baseline.

These results suggest that the model did not learn a globally applicable calibration function for SPR. Instead, it appears to rely on region-specific correction patterns—adapted to the radar geometry, terrain effects, and precipitation characteristics present in the training regions. When exposed to a new geography like Maui, where radar error modes may differ, the model’s learned adjustments no longer generalize reliably. This is especially evident in the drop in recall for heavier rain rates, where the model becomes more conservative in the absence of familiar correction cues.

These findings reinforce that the model learns effective local corrections but does not generalize across unseen regions, suggesting that globally consistent radar calibration remains an open challenge. The full class-wise breakdown is shown in Table 3.

### 3.5. Error Patterns Across Rainfall Classes

**Figure 5:**
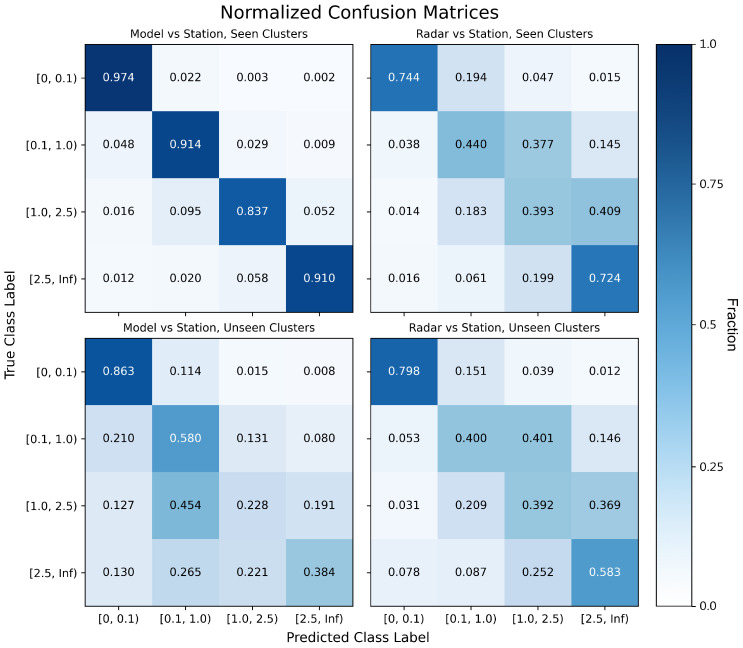
Normalized confusion matrices comparing predicted rainfall class against station-derived labels. Top row: performance on spatial clusters seen during training. Bottom row: performance on the unseen Maui cluster. Left column: predictions from the model. Right column: predictions from NOAA’s SPR radar baseline. Each matrix is row-normalized; diagonal elements represent correct classifications, while off-diagonal elements indicate misclassifications.

While class-wise precision, recall, and F1 scores provide valuable quantitative summaries, they do not reveal the specific nature of the model’s errors—such as whether it tends to overestimate or underestimate rainfall intensity for a specific class. To better understand how predictions differ from ground truth, we present normalized confusion matrices (Figure 5) that visualize classification outcomes across all rainfall intensity categories for both seen and unseen regions.

In each matrix, rows represent the true rainfall classes as derived from rain gauge observations, and columns represent the predicted classes. Values are normalized by row, so each row sums to one and reflects the distribution of predicted categories for a given true class. Diagonal elements indicate correct predictions; off-diagonal elements show misclassifications. Darker shading corresponds to a higher fraction of cases in each cell.

The top row of Figure 5 shows performance on regions seen during training. The model (top left) exhibits strong agreement with the ground truth, with over 90% of predictions falling on the diagonal across all classes—indicating accurate and consistent classification. In contrast, the radar SPR baseline (top right) demonstrates substantial confusion, particularly in the light and moderate categories. It frequently overestimates rainfall intensity, often misclassifying light rain as moderate or heavy.

The bottom row illustrates performance on the unseen Maui cluster. Here, the model (bottom left) maintains superior performance in the low and light rainfall class but shows increased confusion in moderate and heavy categories. It often underestimates rainfall, predicting lower intensities than observed. This behavior is consistent with the drop in recall noted earlier and likely reflects the model’s uncertainty in a region with unfamiliar radar characteristics. The radar baseline (bottom right) performs similarly in both seen and unseen regions, showing the same systematic biases—such as overprediction of light and moderate rainfall—regardless of location.

Overall, these confusion matrices reinforce earlier findings: the model performs well in familiar regions and maintains some skill in new areas, especially for low-intensity rainfall. However, it does not generalize its calibration boundaries for heavier rainfall, and tends to default to more conservative predictions in unfamiliar environments.

## 4. Discussion and Future Work

Our results demonstrate that deep learning models can meaningfully improve radar-based precipitation estimation through localized calibration. On spatial clusters seen during training, the model significantly outperforms NOAA’s operational SPR products, achieving substantial gains in precision, recall, and F1 score across all rainfall classes. These results highlight the model’s ability to learn region-specific radar error patterns and leverage spatiotemporal radar sequences to improve rainfall classification. In operational terms, this suggests that data-driven methods—when informed by historical ground truth—can enhance near-real-time rainfall products in complex terrain and climatologically diverse regions.

However, generalization to unseen spatial clusters remains a clear limitation. On the held-out Maui test set, performance gains relative to the radar baseline are modest and uneven. While the model shows some skill in classifying light and moderate rainfall, recall for heavy precipitation degrades significantly. This reflects the model’s reliance on regional correction patterns rather than a globally consistent calibration strategy. It also highlights the challenge of spatial transfer in radar data, where beam geometry, terrain interference, and precipitation structure can vary sharply between locations. Addressing this limitation might address scaling such models for broader operational use.

Beyond model generalization, label quality introduces another source of uncertainty. Our piecewise-linear accumulation fitting method allowed us to estimate high-frequency rainfall rates from sampled gauge accumulations. While effective in producing millions of training labels, this method remains sensitive to the inherent noise in PWS data. The lack of standardized instrumentation and the discretized nature of tipping-bucket gauges introduce residual error in label construction. Future research should explore alternative accumulation modeling techniques—such as spline-based interpolation or constrained non-linear regression. Better accumulation models could reduce label noise and improve learning signal, particularly in edge cases involving light or intermittent rainfall.

Another important direction is a systematic comparison of deep learning architectures for radar-based precipitation classification. While our use of a ResNet-101 backbone yielded strong performance with relatively low inference latency, other architectures—such as ConvLSTMs, 3D CNNs, or Transformer-based models—may offer different tradeoffs between spatial–temporal expressiveness and computational efficiency. A structured evaluation of these architectures, benchmarked across accuracy, generalization, and runtime cost, would provide valuable guidance for both research and operational deployment. This would be particularly relevant for real-time forecasting systems, where latency constraints are often as critical as predictive performance.

On the modeling side, incorporating additional input modalities—such as satellite precipitation estimates, numerical weather prediction model fields, or surface meteorological observations—could help provide broader contextual signals and improve generalization to unfamiliar environments. Coupling such data integration with uncertainty estimation mechanisms would allow models not only to predict rainfall intensity but to quantify confidence in those predictions.

While challenges remain, this work demonstrates a viable approach for enhancing real-time rainfall estimation by leveraging historical citizen science observations and spatiotemporal radar patterns. With further advances in label quality, model architecture, and generalization capability, deep learning offers a compelling pathway toward more accurate, faster, and more adaptable precipitation products for forecasting and emergency response.

## 5. Conclusions

This study demonstrates that deep learning can substantially improve radar-based precipitation estimation by learning localized calibration corrections from historical citizen science observations. By framing rainfall prediction as a classification problem, we achieved significant improvements over NOAA’s operational SPR product within regions seen during training. These gains emphasize the value of integrating dense, ground-level observations into radar correction pipelines, even when those observations are collected from non-professional networks. At the same time, the model’s limited performance in unseen regions highlights the challenges of spatial generalization in radar-based rainfall prediction. This underscores the need for improved label construction, expanded geographic training diversity, and architectures that can better adapt to regional variability. Overall, our findings point to a promising future for data-driven precipitation estimation. With continued advances in observational datasets, model design, and integration with operational systems, deep learning has the potential to enhance both the accuracy and responsiveness of real-time rainfall monitoring and short-term forecasting.

## Figures and Tables

**Table 1:** Data splits per cluster. Clusters 1–8 are used for training, validation, and testing; 9 is held out for final testing only.

Cluster	Island	Station Count	Cluster RQI	Data Entries	Testing Allocation
1	O’ahu	18	Low	60,631	30%
2	Hawai’i	67	Moderate	363,745	30%
3	O’ahu	59	Moderate	279,670	30%
4	O’ahu	51	Moderate	387,817	30%
5	Kaua’i	14	High	66,727	30%
6	Hawai’i	13	High	30,607	30%
7	Kaua’i	16	Moderate	35,125	30%
8	Hawai’i	18	Low	85,378	30%
9	Maui	49	Low	470,603	100%

**Table 2:** Generalization within observed spatial clusters. Note that higher values are better.

	Our Model			Radar		
Class	F1 Score	Precision	Recall	F1 Score	Precision	Recall
[0, 0.1)	0.968	0.963	0.974	0.838	0.956	0.744
[0.1, 1.0)	0.913	0.912	0.914	0.471	0.506	0.440
[1.0, 2.5)	0.845	0.853	0.837	0.230	0.236	0.393
[2.5, *∞*)	0.921	0.932	0.910	0.608	0.524	0.724
Macro	**0.912**	**0.915**	**0.909**	0.553	0.556	0.575

**Table 3:** Generalization to unseen spatial clusters. Note that higher values are better.

	Our Model			Radar		
Class	F1 Score	Precision	Recall	F1 Score	Precision	Recall
[0, 0.1)	0.869	0.874	0.863	0.867	0.953	0.798
[0.1, 1.0)	0.538	0.501	0.580	0.422	0.445	0.400
[1.0, 2.5)	0.241	0.255	0.228	0.266	0.202	0.392
[2.5, *∞*)	0.427	0.482	0.384	0.500	0.437	0.583
Macro	**0.519**	**0.528**	0.514	0.514	0.509	**0.543**

**Figure A1:**
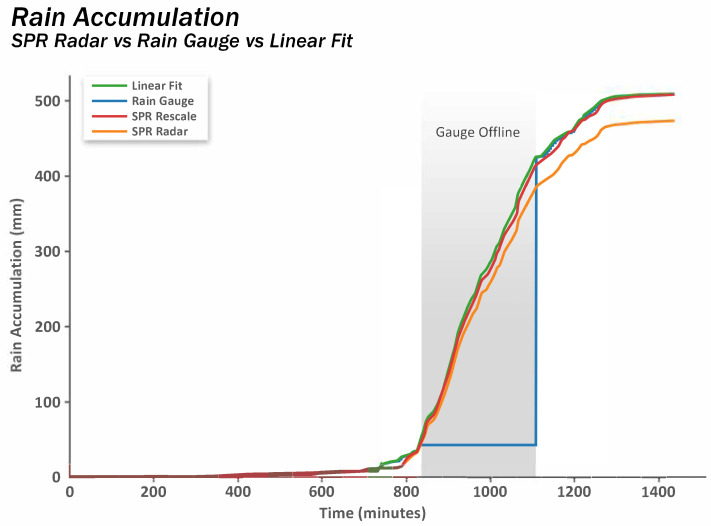
Example of a rain gauge that was temporarily offline but still produced usable accumulation information after reactivation.

## Data Availability

The dataset is available at https://www.kaggle.com/datasets/rosenhoover/hawaii-radar-to-rain-gauge-rain-rate-prediction. The Code for training the model is available at https://github.com/Marshall-Rosenhoover/Improving-Doppler-Radar-Precipitation-Prediction-with-Deep-Learning-and-Citizen-Science-Rain-Gauges.

## Appendix A. Details on Accumulation Function Construction

### Appendix A.1. Gauge Sensitivity and Reference Level Selection

A key assumption in reconstructing accumulation functions is how to interpret each reported gauge value $O_{i}$, which only guarantees that the true accumulation lies within a range defined by the gauge sensitivity *m*. To linearly rescale radar-derived accumulation within each interval, a decision must be made about where within this uncertainty range to anchor the true value.

We evaluated three possible reference levels: the lower bound (floor), the midpoint, and the upper bound (ceiling). Empirically, anchoring to the floor produced the most consistent and physically plausible results. This choice conservatively respects the quantization limits of each gauge and avoids introducing artificial rainfall signals during low-intensity or near-zero events. While more complex interpolation methods are possible, the linear rescaling approach offers simplicity and stability given the resolution and noise characteristics of the data.

### Appendix A.2. Benefits and Limitations of the Linear Rescaling Approach

The piecewise-linear accumulation model assumes that rainfall within each observation interval follows the temporal pattern of radar-derived rain rates, scaled to match the gauge’s accumulated total. While this introduces potential inaccuracies during periods of radar error or missing gauge data, the approach offers several practical advantages.

First, because the adjustment is performed independently within each gauge interval, the method remains robust to irregular reporting. If a gauge is temporarily offline and later comes back online with a large accumulated total, the method can still produce a valid reconstruction. Figure A1 shows an example of such a case. This interval-based structure is particularly well suited to the uneven and sometimes sparse reporting patterns of citizen science networks.

### Appendix A.3. Permissible Cases of Gauge Decrease

While rainfall accumulation is generally expected to be non-decreasing, small drops in reported values can occur due to two common and physically plausible phenomena:

- Gauge Emptying: Many personal weather stations periodically empty accumulated water, resetting the gauge reading to zero. This typically occurs as part of routine maintenance or automated station functions.
- Evaporation Loss: During dry periods, small evaporation losses can reduce the measured water level enough to lower the recorded accumulation by one sensitivity increment.

Our implementation detects such drops and reinitializes the accumulation function accordingly. To maintain consistency, any subsequent accumulation after a drop is anchored using the floor of the new observation range, in line with our general rescaling strategy. This conservative handling avoids introducing false rainfall during dry periods or underrepresenting rainfall after a reset.

However, this approach does not attempt to model evaporation loss within an observation interval. A more sophisticated formulation could account for potential evaporation between gauge reports—adjusting the accumulation curve accordingly. For example, such a method might scale radar-derived rainfall rates not just to match the total increase, but to also account for the fraction of rainfall that may have evaporated before being recorded. Incorporating such physical loss mechanisms remains an interesting direction for future work, particularly in dry climates or low-intensity events where evaporation effects may be non-negligible.

### Appendix A.4. Handling Radar–Gauge Disagreements

One challenge in constructing accumulation functions arises when radar and gauge observations disagree—specifically, when one indicates rainfall and the other does not. This can occur in two directions:

- Gauge increase with zero radar: If the gauge reports a rise in accumulation but radar estimates are zero throughout the interval, the entire increase must be assigned to the final time step in that interval. This produces an artificially high instantaneous rain rate at the end of the window to satisfy the gauge constraint.
- Radar rain with static gauge: Conversely, if radar indicates nonzero rainfall but the gauge shows no accumulation change, our method assigns a rain rate of zero throughout the interval. This behavior results from our decision to align each gauge reading to the floor of its sensitivity range, which inherently prioritizes physical consistency with the gauge measurement.

These cases represent a fundamental limitation of reconciling sparse or quantized ground-truth data with continuous radar observations. Our conservative approach avoids introducing spurious rain where the gauge reports none and ensures that total accumulation is never overstated. However, it may underrepresent actual rain intensity in cases where radar detects light precipitation that does not exceed the gauge’s sensitivity threshold.

More advanced methods could attempt to distribute gauge-derived accumulation more flexibly within the interval, even in the presence of apparent radar–gauge disagreement. For example, smoothing functions or probabilistic models could account for partial radar support without violating gauge constraints. Nevertheless, in practice, such disagreements were relatively rare and often limited to short, low-intensity intervals. As such, their overall impact on model training is likely minimal—but remains a valuable consideration for future refinement.
